# Fluorescence contrast-enhanced proliferative lesion imaging by enema administration of indocyanine green in a rat model of colon carcinogenesis

**DOI:** 10.18632/oncotarget.21744

**Published:** 2017-10-09

**Authors:** Nobuhiko Onda, Reiko Mizutani-Morita, Susumu Yamashita, Rei Nagahara, Shinya Matsumoto, Toshinori Yoshida, Makoto Shibutani

**Affiliations:** ^1^ Evaluation Technology Department 1, R&D Group, Olympus Corporation, Hachioji, Tokyo 192-8512, Japan; ^2^ Laboratory of Veterinary Pathology, Division of Animal Life Science, Institute of Agriculture, Tokyo University of Agriculture and Technology, Fuchu, Tokyo 183-8509, Japan; ^3^ Institute of Global Innovation Research, Tokyo University of Agriculture and Technology, Fuchu, Tokyo 183-8509, Japan

**Keywords:** colon tumor, endoscopy, fluorescence imaging, indocyanine green, tight junction

## Abstract

The fluorescent contrast agent indocyanine green (ICG) is approved by the Food and Drug Administration for clinical applications. We previously reported that cultured human colon tumor cells preferentially take up ICG by endocytic activity in association with disruption of their tight junctions. The present study explored ICG availability in fluorescence imaging of the colon to identify proliferative lesions during colonoscopy. The cellular uptake of ICG in cultured rat colon tumor cells was examined using live-cell imaging. Colon lesions in rats administered an ICG-containing enema were further assessed in rats with azoxymethane-induced colon carcinogenesis, using *in vivo* endoscopy, *ex vivo* microscopy, and immunofluorescence microscopy. The uptake of ICG by the cultured cells was temperature-dependent. The intracellular retention of the dye in the membrane trafficking system suggested endocytosis as the uptake mechanism. ICG administered via enema accumulated in colon proliferative lesions ranging from tiny aberrant crypt foci to adenomas and localized in proliferating cells. Fluorescence endoscopy detected these ICG-positive colonic proliferative lesions *in vivo*. The immunoreactivity of the tight-junction molecule occludin was altered in the proliferative lesions, suggesting the disruption of the integrity of tight junctions. These results suggest that fluorescence contrast-enhanced imaging following the administration of an ICG-containing enema can enhance the detection of mucosal proliferative lesions of the colon during colonoscopy. The tissue preference of ICG in the rat model evaluated in this study can be attributed to the disruption of tight junctions, which in turn promotes endocytosis by proliferative cells and the cellular uptake of ICG.

## INTRODUCTION

Colorectal cancer is the second most common cancer in women and the third most common in men [1]. Currently, screening colonoscopy is widely used as the gold standard for the detection of colorectal cancer, as the early detection of polyps followed by their endoscopic removal has been shown to prevent the development of colorectal cancer and thus mortality due to the disease [2, 3]. However, this strategy has several limitations with respect to the detection of small lesions and the microscopy-based identification of their pathological features. In fact, up to 25% of the polyps in the colon will be missed by white-light endoscopy [4], which relies on visual inspection to detect structural abnormalities. The development of diagnostic techniques that provides molecular information with sufficient sensitivity and specificity would allow improved early cancer detection and thus earlier intervention.

Fluorescence tumor imaging using exogenous fluorescent tumor-targeting agents has been investigated for applications in endoscopy and surgery [5–8]. In this technique, neoplastic lesions are visually enhanced with specific molecular markers targeted by the fluorescent agents. The advantages of fluorescence tumor imaging include its high specificity and sensitivity, real-time video frame-rate imaging, relatively low cost, portability, the absence of radiation exposure, and the option of simultaneous multiplexed imaging using fluorescent agents differing in their wavelengths [9, 10]. It may therefore be a valuable method in early cancer detection and in minimally invasive image-guided surgical procedures. However, despite the variety of tumor imaging agents developed thus far, many of those intended for use in humans are still in pre-clinical or early-phase clinical trials [6].

The fluorescent contrast agent indocyanine green (ICG) has been clinically approved by the Food and Drug Administration for human applications and has an excellent safety profile in hepatic function tests [11, 12]. Recently, the utility of intravenously administered ICG in tumor detection in a variety of tumor types was reported in experimental models [13–18] and in clinical cancers [19–28]. In hepatic tumors, ICG positivity correlates in part with the expression of the membrane transporters organic anion transporting polypeptide 1B3 (OATP1B3), and sodium-taurocholate co-transporting polypeptide (NTCP) [21, 26]. Both are normally expressed in hepatocytes and mediate the cellular uptake of ICG *in vitro* [29].

We recently reported that cultured-human colon tumor cells incubated with ICG take up the dye via the endocytic pathway, in parallel with the activity of membrane transporters [18]. The endocytic activity was associated with disruption of the tight junctions (TJs) of the tumor cells [18]. Both an abnormally high endocytic rate and the loss of TJs are hallmarks of cancer [30, 31], and the altered expression of TJ molecules during colon tumor progression has been reported in clinical cancers [31–33] and in experimental models [34]. These studies suggest that TJ disruption in colon tissues can serve as a cell-surface biomarker in ICG fluorescence imaging.

The aim of the present study was to investigate the utility of ICG topically applied to the colon surface in the imaging of proliferative lesions of the colon. Thus, rats with azoxymethane (AOM)-induced colon cancer were administered an ICG enema for 30 min after which *in vivo* endoscopy and *ex vivo* microscopy images were obtained. In addition, the mechanism underlying the cellular uptake of ICG was examined in cultured rat colon tumor cells.

## RESULTS

### ICG uptake in cultured rat colon tumor cells

**Figure 1:**
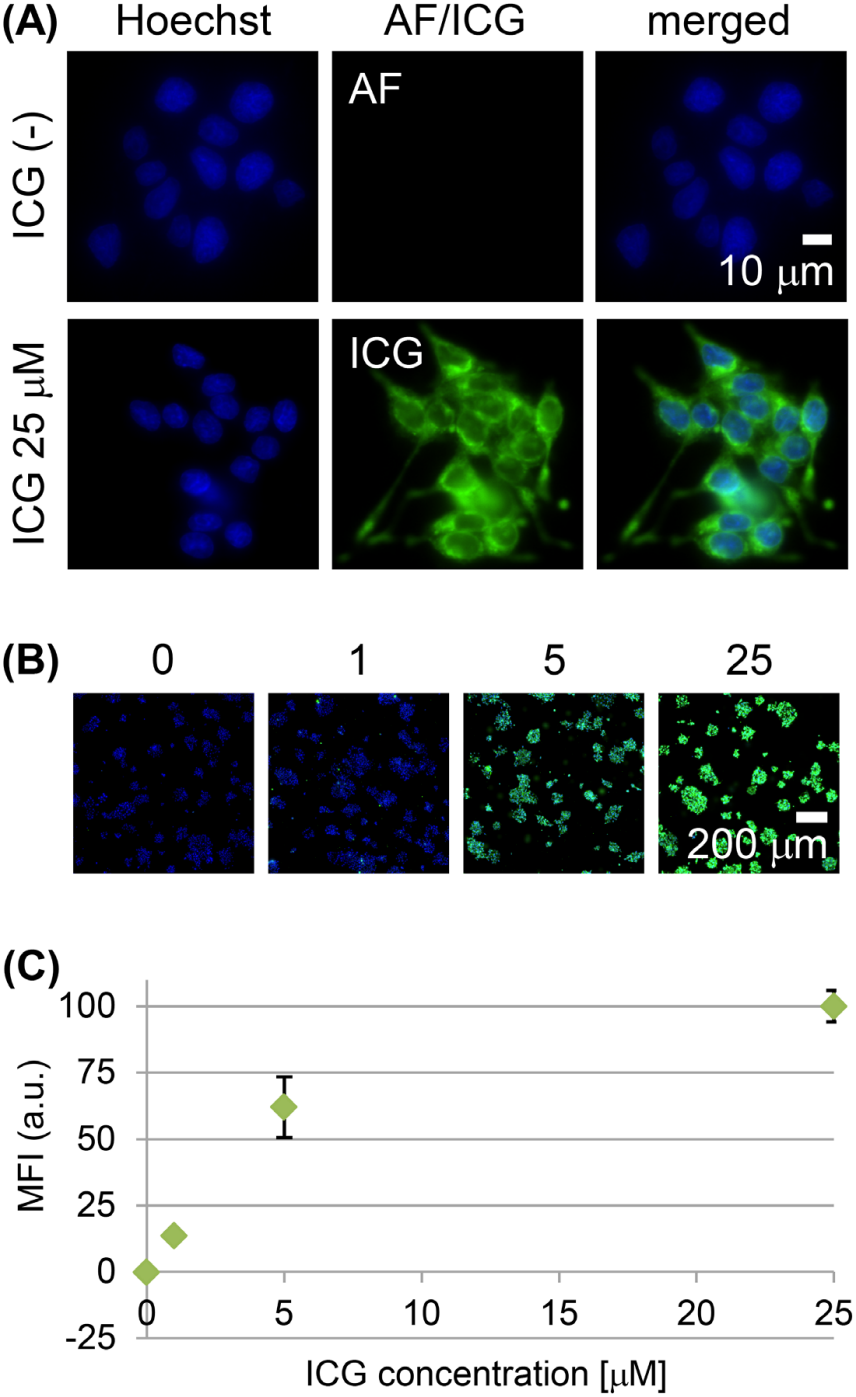
Live-cell imaging of RCN-9 cells incubated with indocyanine green (ICG) **(A)** High-power view of RCN-9 cells incubated in the presence or absence of ICG (green) at 37°C for 30 min, followed by Hoechst 33342 (blue) for nuclear staining. AF: autofluorescence. Scale bar, 10 μm. **(B)** Whole-scan view of RCN-9 cells incubated with 0, 1, 5, or 25 μM ICG (green) at 37°C for 30 min, followed by Hoechst 33342 (blue). Scale bar, 200 μm. **(C)** Mean fluorescence intensity (MFI) of ICG-labeled RCN-9 cells per whole-scan view. Error bars represent means ± SD.

**Figure 2:**
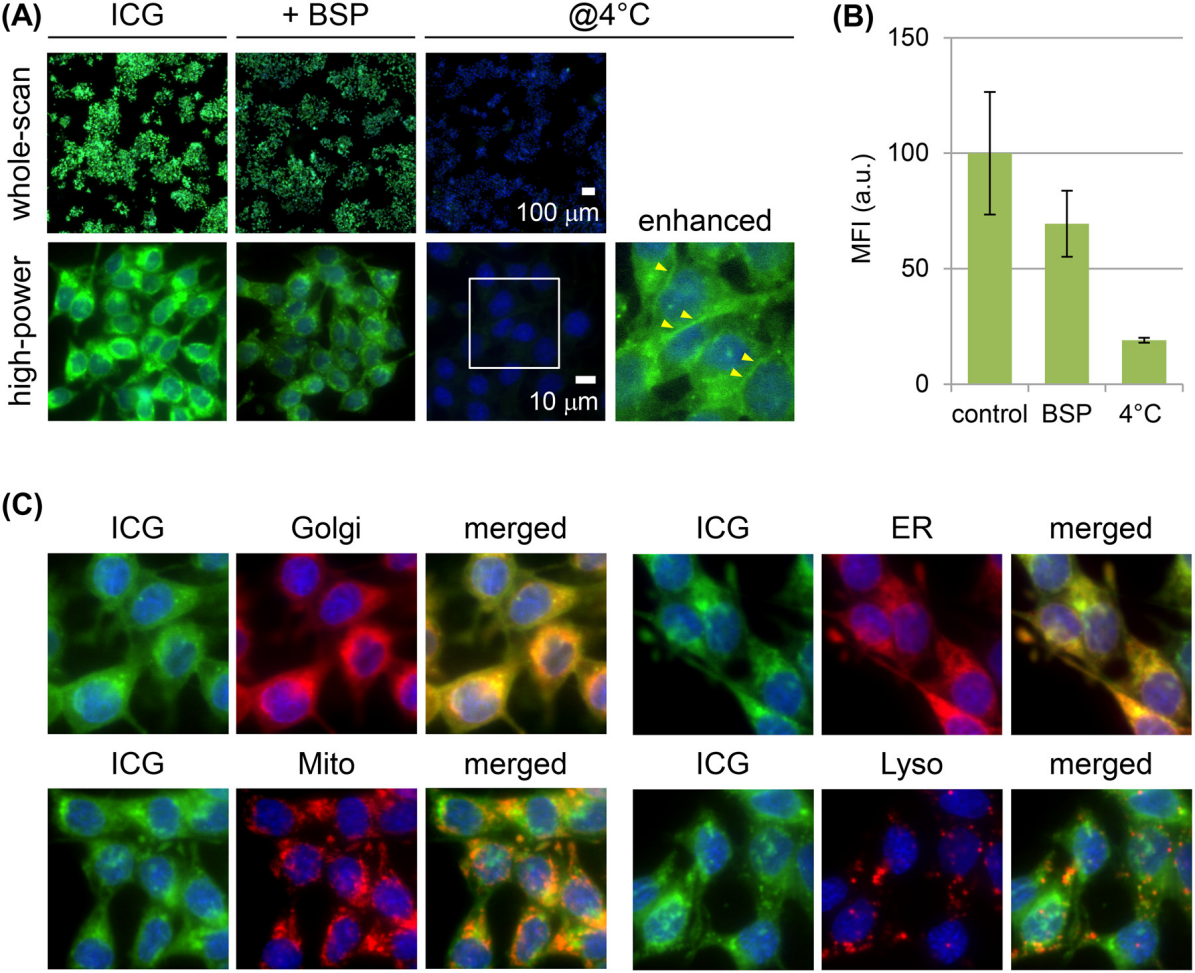
Mechanism of ICG uptake by RCN-9 cells **(A)** Live-cell imaging of RCN-9 cells incubated for 30 min with ICG (green) in the presence of the membrane transporter inhibitor sulfobromophthalein (BSP) or at 4°C, followed by incubation with Hoechst 33342 (blue). Upper panels: whole-scan view. Scale bar, 100 μm. Lower panels: high-power view. Live cell images as shown in the boxed areas indicate the field-of-view of the magnified and enhanced images. The arrowhead indicates the ICG fluorescence signal at the plasma membrane. Scale bar, 10 μm. **(B)** Mean fluorescence intensity (MFI) of ICG-labeled RCN-9 cells per whole-scan view. Error bars represent means ± SD. **(C)** Live-cell imaging of RCN-9 cells stained for organelles (red) and nuclei (blue) after a 30-min incubation with ICG (green). Golgi: Golgi apparatus; ER: endoplasmic reticulum; Mito; mitochondria; Lyso; lysosome.

Cultured RCN-9 rat colon tumor cells showed weak cell-cell adherence and did not completely form epithelioid-structure. RCN-9 cells incubated for 30 min with ICG had a detectable intracellular ICG fluorescence signal, in contrast to the cells without ICG incubation (Figure 1A), and the signal increased in a dose-dependent manner (Figure 1B, 1C). Incubation of RCN-9 cells with sulfobromophthalein (BSP) only partially inhibited the cellular uptake of ICG (Figure 2A, 2B). However, at 4°C uptake was almost completely inhibited, with weak ICG fluorescence at the plasma membrane as well as in the cytoplasm (Figure 2A, 2B). After a 30-min incubation with ICG at a temperature of 37°C, intracellular ICG fluorescence was seen in the Golgi-endoplasmic reticulum system and partially in mitochondria and lysosomes (Figure 2C).

### Tumor uptake of ICG administered via enema to rats in a model of colon carcinogenesis

**Figure 3:**
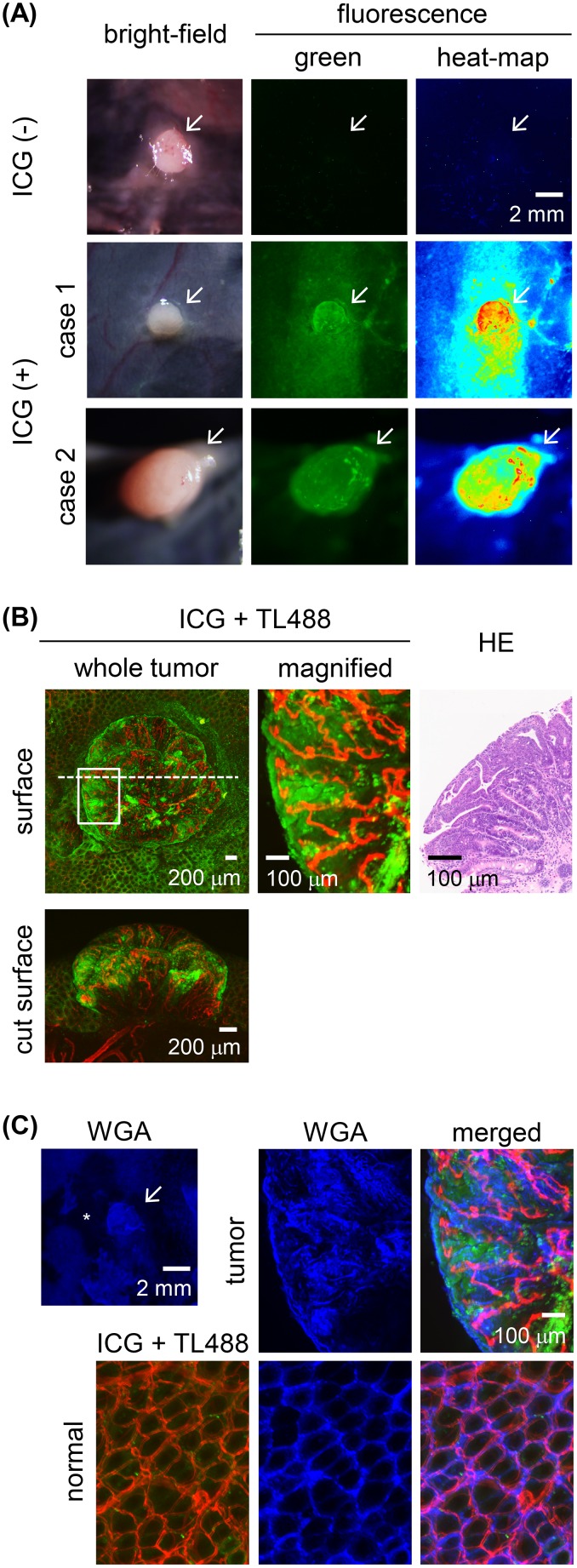
*Ex vivo* ICG-fluorescence imaging of colon tumors in azoxymethane (AOM)-induced colon carcinogenesis in rats after a 30-min ICG enema **(A)** Wide-field images of colon adenoma tissues incubated in the presence or absence of ICG. Left panels: bright-field images. Middle panels: fluorescence images with pseudo-colored green. Right panels: fluorescence images with pseudo-colored heat map. Representative ICG^+^ colon adenomas are shown in case 1 and case 2. Colon adenoma in case 2 was developed at the position next to the cecum. Arrow: colon adenoma. Scale bar, 2 mm. **(B)** High-power view of the ICG^+^ (green) colon adenoma shown in the case 1 of the panel (A). Fluorescence images of the tumor surface as shown in the boxed areas and by the dashed line indicate the field-of-view of the magnified image and the tumor cut plane, respectively. Blood vessels were stained with *Lycopersicon esculentum* (tomato) lectin conjugated with DyLight 488 (TL488; red). Image of a hematoxylin & eosin (HE)-stained section of the colon tumor corresponding to the *ex vivo* image. Scale bar for the whole-tumor view, 200 μm; for the magnified view, 100 μm. **(C)** Fluorescent wheat germ agglutinin (WGA; blue) stained images of ICG^+^ (green) colon adenoma tissues shown in the case 1 of the panel (A). Blood vessels were stained with TL488 (red). Asterisk indicates the area where mucus was removed during staining procedure. Scale bar for the whole-tumor view, 2 mm; for the magnified view, 100 μm.

In the four animals not administered an ICG enema, weak autofluorescence signals were detected in four adenomas and one adenocarcinoma and in the normal colon (Figure 3A). The four adenomas showed preferential uptake of ICG after the administration of a 30-min ICG enema to the rats. The fluorescence was distributed heterogeneously in the tumor tissues (Figure 3A), specifically, in their tubular epithelia and forming a tubular gland pattern (Figure 3B). Vertical distribution of the ICG fluorescence, localized only at the colon tumor surface, was also observed (Figure 3B). Fluorescence signal of wheat germ agglutinin (WGA), which binds to glycoproteins and is used for mucus imaging [35], was observed both in the tumor tissues and normal colonic mucosa (Figure 3C).

### Immunoreactivity of TJ proteins and membrane transporters in rat colon tumor tissues

**Figure 4:**
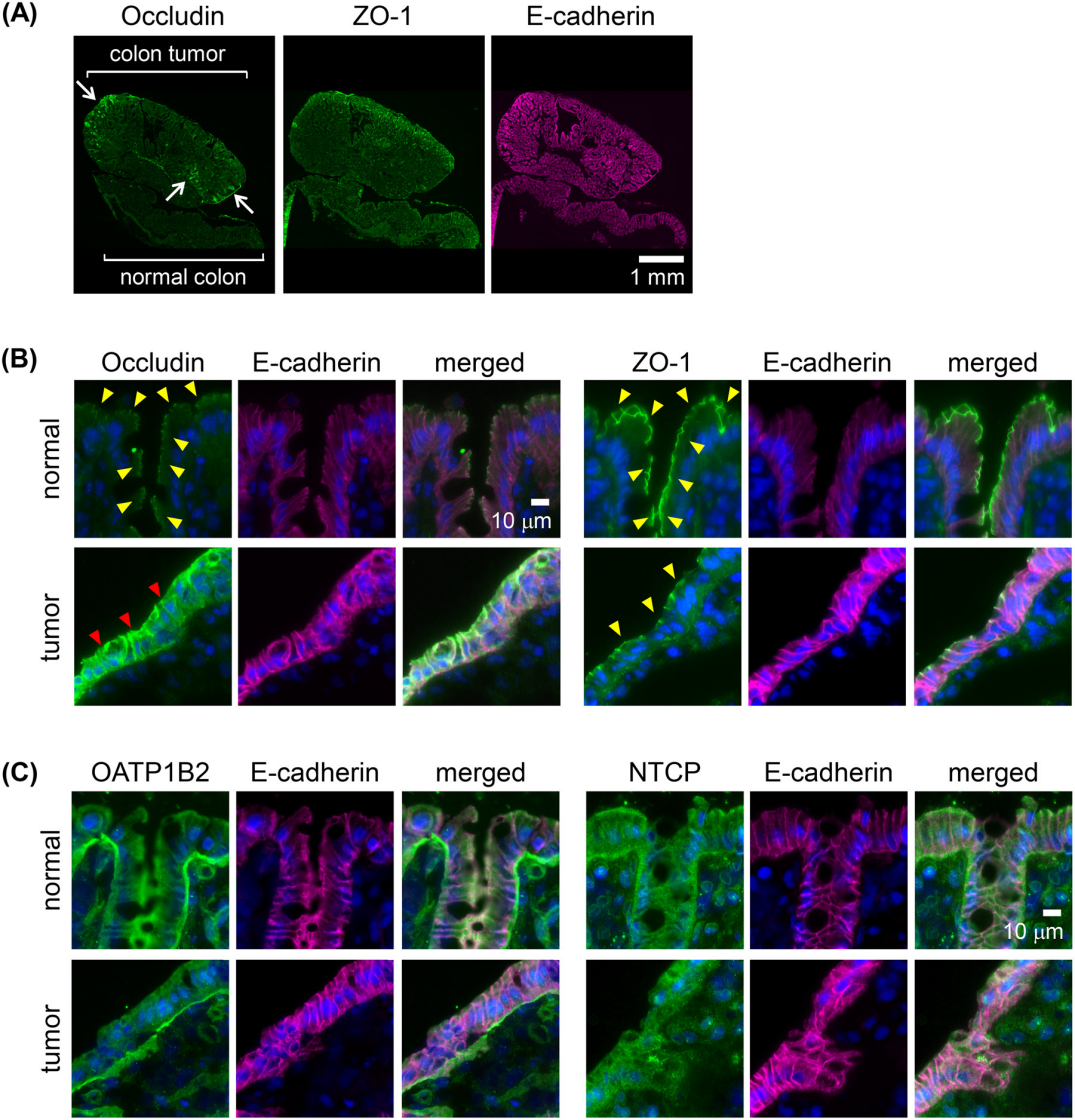
Immunoreactivities of membrane transporters and tight junction (TJ) molecules in AOM-induced colon carcinogenesis in rats **(A)** Whole-tissue view of colon adenoma tissues with adjacent normal colonic epithelia immunostained for the TJ molecule zona occludens-1 (ZO-1) or occludin (green) and E-cadherin (adherence junction marker as a reference; magenta). Arrows: strong signals of occludin. Scale bar, 1 mm. **(B)** High-power view of normal colonic epithelium or colon adenoma tissues double-immunostained with E-cadherin (magenta) and ZO-1 or occludin (green), with nuclear staining (blue). Yellow arrowheads: signals at the apical cell border. Red arrowheads: strong signals distributed in the lateral cell walls and apical cell border. Scale bar, 10 μm. **(C)** High-power view of normal colonic epithelium or colon adenoma tissues double-immunostained with E-cadherin (magenta) and for the membrane transporters organic anion transporting polypeptide 1B2 (OATP1B2) or sodium-taurocholate co-transporting polypeptide (NTCP) (green), with nuclear staining (blue). Scale bar, 10 μm. All two adenomas and two adenocarcinomas showed similar results.

The immunofluorescence of the two adenomas and two adenocarcinomas as well as their adjacent normal tissues was analyzed. Occludin, a key molecule in TJ formation at the apical cell border, was expressed at the site of TJs of the normal colonic epithelium (Figure 4A, 4B). By contrast, in the adenomas and adenocarcinomas, occludin was heterogeneously overexpressed in multifocal areas, including the colon tumor surface (Figure 4A). In the areas of overexpression, occludin was detected in the tumor cells both at the lateral plasma membrane and at the apical cell border (Figure 4B). The immunoreactivity of ZO-1, another key molecule involved in TJ formation, was localized at the apical cell border both in normal colonic epithelia and in the adenomas and adenocarcinomas (Figure 4A, 4B). For the membrane transporters OATP1B2 [36], the rat ortholog of human OATP1B1 and OATP1B3, and NTCP, their immunoreactivity in adenomas and adenocarcinomas of the rat colon did not differ from that of normal colonic epithelial cells (Figure 4C).

### Endoscopic imaging of rat colon tumors after ICG administered via enema

**Figure 5:**
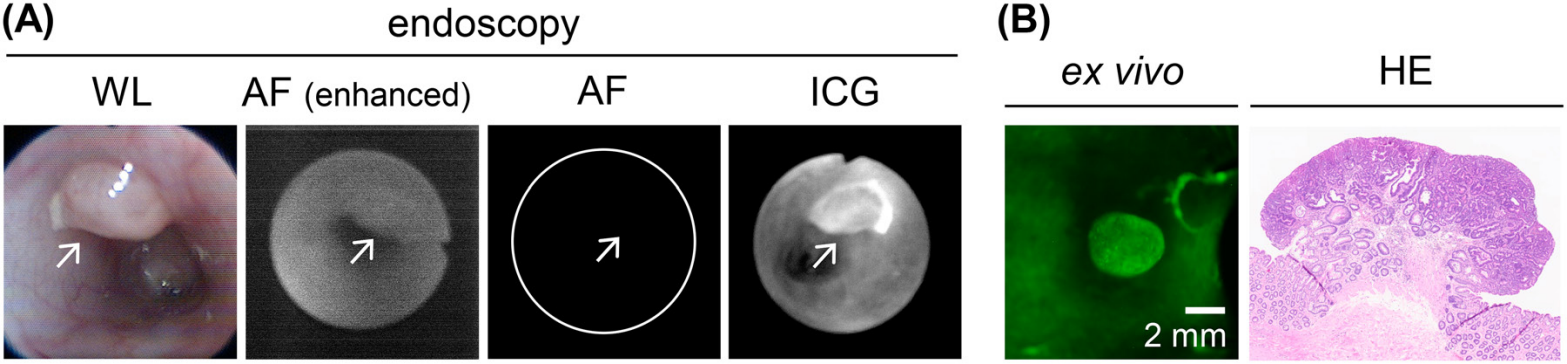
*In vivo* endoscopic imaging of colon tumors in AOM-induced colon carcinogenesis in rats after a 30-min ICG enema **(A)** *In vivo* endoscopy image of ICG^+^ colon adenoma tissues. Left panel: white-light (WL) image. Left middle panel: autofluorescence (AF) image before the ICG enema. The fluorescence signal is enhanced to show tissue morphology. Right middle panel: autofluorescence image before the ICG enema. The fluorescence signal is shown using the same conditions as the fluorescence image after the ICG enema. Right panel: fluorescence image after the ICG enema. Arrow: colon tumor. **(B)** *Ex vivo* ICG-fluorescence image and HE-stained section image of the colon adenoma. The images correspond to the endoscopic images as shown in (A). Scale bar, 2 mm.

White-light endoscopy revealed tumor nodules in the rat colon (Figure 5A, 5B). After switching to fluorescence imaging, *in vivo* autofluorescence signals in the colon tumors and normal colon were rarely seen (Figure 5A, 5B). After the administration of an ICG-containing enema for 30 min, *in vivo* fluorescence endoscopy showed increased ICG fluorescence of the colon tumors (Figure 5A, 5B).

### ICG fluorescence imaging of small proliferative lesions

**Figure 6:**
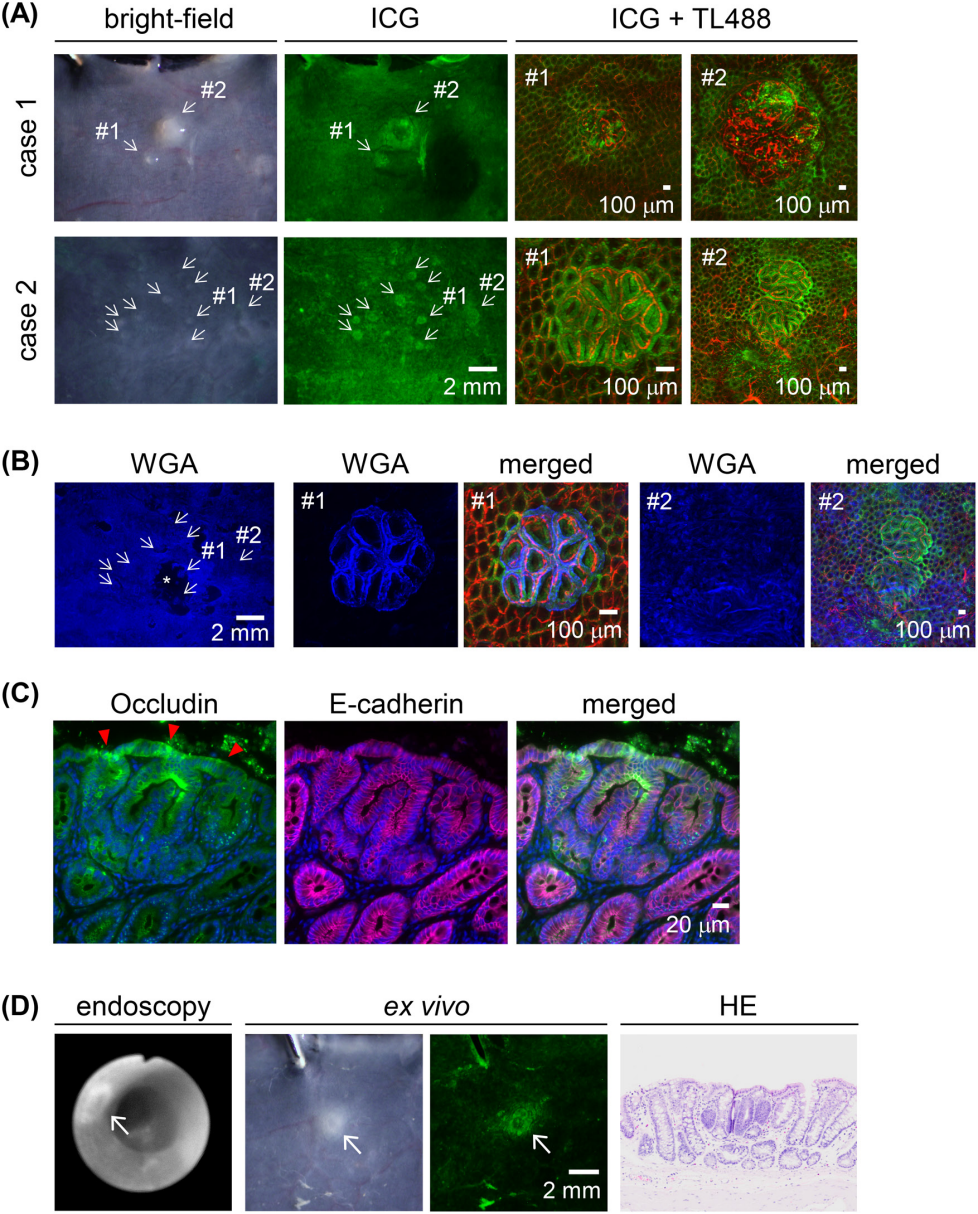
ICG fluorescence imaging of macroscopically small proliferative lesions in AOM-induced colon carcinogenesis in rats after a 30-min ICG enema **(A)** *Ex vivo* images. Left panels: bright-field images. Middle panels: ICG fluorescence (green) images. Small proliferative lesions are indicated by arrows in both case 1 and case 2. Scale bar, 2 mm. Right panels: representative high-power views of small proliferative lesions with blood vessel staining (TL488; red). In case 1, both #1 and #2 were histologically diagnosed as aberrant crypt foci (ACF); in case 2, #1 was diagnosed as adenoma and #2 as focal mucosal hyperplasia. Scale bar, 100 μm. **(B)** Fluorescent WGA (blue) stained images of ICG^+^ (green) proliferative lesions in the case 2 of the panel (A). Blood vessels were stained with TL488 (red). Asterisk indicates the area where mucus was removed during staining procedure. Scale bar for the whole-tumor view, 2 mm; for the magnified view, 100 μm. **(C)** ACF double-immunostained with occludin (green) and E-cadherin (magenta), with nuclear staining (blue). Red arrowheads: strong signals distributed in the lateral cell walls and apical cell border. Scale bar, 20 μm. **(D)** *In vivo* imaging of ACF by fluorescence endoscopy. *Ex vivo* bright-field and ICG-fluorescence image and HE-stained section image corresponding to the endoscopic images. Arrow: ACF. Scale bar, 2 mm.

Small proliferative lesions, including focal mucosal hyperplasia and aberrant crypt foci (ACF), were sometimes difficult to identify on the white-light images but on fluorescence imaging showed the preferential uptake of ICG after the 30-min administration of the ICG-containing enema (Figure 6A). Fluorescent WGA signal was observed both in the proliferative lesions and normal colonic mucosa (Figure 6B). The altered occludin immunoreactivity in the ACF lesions was similar to that in the colon tumor tissues (Figure 6C). Fluorescence endoscopy revealed the ACF lesions, which were < 1 mm in diameter, 30 min after ICG exposure (Figure 6D).

## DISCUSSION

This study demonstrated the ability of topical ICG application to reveal colon tumor tissues throughout the colon in a rat model of colon cancer. A previous study showed that intravenously administered ICG preferentially accumulates in xenograft tumors consisting of human colon tumor cells [18]. Similar results were obtained in clinical cancers, including colorectal carcinomatosis [19, 22] and their lymph node [23] or liver [20] metastases. In other recent studies using *ex vivo* disseminated peritoneal nodules from clinical digestive cancer, topical application of ICG visualized the micro-structures of the tumor surface [37] and clusters of cancerous cells [38], as seen using probe-based confocal laser endomicroscopy with a high-power albeit small field-of-view. To the best of our knowledge, ours is the first report in which topically applied ICG was used in the *in vivo* identification of tiny proliferative lesions as well as tumors during colonoscopy, aided by its wide field-of-view.

In the present study, we used a rat model of colon carcinogenesis, AOM-induced colon cancer, and visualized the tumors by endoscopy. The polypoid architecture and molecular and clinical features of these tumors are similar to those of sporadic human colorectal cancers [39, 40]. The AOM model also leads to the development of preneoplastic lesions (ACF). Therefore, this model was suitable to evaluate the topical application of ICG in colonoscopy, in contrast to the ectopic xenograft tumor model used in a previous study [18]. Experiments in cultured cells were carried out using the RCN-9 cell line, established from a 1,2-dimethylhydrazine (DMH)-induced rat colon tumor [41]. Because DMH is a precursor of AOM, the two compounds share several metabolic activation steps to induce DNA-reactive adducts, resulting in colon carcinogenesis [39, 40]. Cultured RCN-9 cells treated with DMH were therefore used in our *in vitro* model, as the counterpart of AOM-induced colon carcinogenesis in rats, to investigate the molecular mechanism underlying preferential ICG accumulation in tumor cells.

Cultured RCN-9 cells used in the current study showed weak cell-cell adherence and did not completely form epithelioid-structure, suggesting an incomplete TJ formation. In accordance with the morphological characteristics, these cells were able to take up ICG, which then became localized in the membrane traffic system. Inhibition of this response by low-temperature incubation suggested endocytosis as the mechanism of uptake, similar to observations in human colon tumor cells [18]. Although ICG was previously shown to be taken up by cells via the membrane transporters OATP1B3 and NTCP [29], in the present study, BSP, a competitive inhibitor of OATPs and NTCP, only partially inhibited the uptake of ICG by cultured RCN-9 cells. Thus, the main mechanism of ICG cellular uptake in RCN-9 cells is endocytosis, with only a limited contribution of membrane transporters.

ICG administered via enema preferentially accumulated in colon tumor tissues and localized in the tumor cells. The immunoreactivities of OATP1B2 [36], a rat ortholog of OATP1B3, and NTCP did not differ between colon tumor and normal colon tissues, which provided further support for the minor role of these proteins in ICG-mediated tumor enhancement, while expression of these membrane transporters in both colon tumor and normal colon tissues may cause increase in the background intensity. However, the expression of occludin was altered, as the protein's distribution was heterogeneous in tumor cells, with strong accumulation in the lateral walls of the cells as well as apically, rather than the apical distribution in normal cells. By contrast, the expression of ZO-1 was normal. A previous study showed that the high endocytic activity of tumor cells in association with the disruption of their TJs accounts for tumor uptake of ICG [18]. In previous work, ICG uptake was higher in the human colon cancer cell line DLD-1, characterized by the altered immunolocalization of occludin in the lateral wall and apical border of the cells but with normal immunoreactivity of ZO-1 at the apical cell border [42], than by T84 cells [18], in which occludin and ZO-1 are expressed normally. The similarly altered expression of occludin in association with disrupted TJs and the normal expression of ZO-1 in the mouse small intestine were also described [43]. These data suggest that the accumulation of ICG by tumor cells in the AOM model of colon cancer in our study can be attributed to disruption of the TJs of tumor cells, leading to their increased endocytic activity and thus an increase in the cellular uptake of ICG.

We also showed that the colon tumor tissues of rats administered an ICG enema could be imaged successfully by *in vivo* endoscopic imaging. To deliver ICG or any other agent to colon tumor tissues for the purpose of imaging, both topical and systemic routes are possible [44]. The advantages of the topical route are the reduced delivery time of the agent to the tumor and the lower dose of the agent while ensuring adequate fluorescence brightness. Although mucus coverage of colonic mucosa may affect ICG delivery to the epithelial cells, our results revealed that mucus detected by fluorescent WGA signal was observed both in the tumor tissues and normal colonic mucosa in the current model, suggesting that ICG uptake after enema administration *in vivo* was not affected by mucus abundance. Furthermore, since fluorescence colonoscopy using different type of fluorescent agent by topical administration [45, 46], such as by enema administration [47], as well as chromo-endoscopy using biologically applicable dyes [48, 49], has already been tried or adopted in the clinical setting, the topical application of ICG can be incorporated into the clinical endoscopic procedure, after optimization of ICG imaging conditions. Although the penetration of ICG was limited at the colon tumor surface 30 min after the enema, longer retention time is impractical in the clinical setting. The low penetrability of ICG is a potential limitation of this approach because it only allows the detection of ICG accumulation in colon tumor epithelia, in which altered molecular expression occurs at the surface of the malignant cells.

ICG administered via enema can label various-sized proliferative lesions, including ACF. In a previous study, the increased permeability of the TJs of the colonic epithelium and the resulting decreased epithelial barrier function preceded the development of colonic tumors [50]. Our method detected the altered expression of occludin even in tiny proliferating lesions, similar to the findings in colon tumor tissues. Together they suggest that TJ disruption is also the molecular event underlying the colonic proliferative lesions that give rise to ACF. Therefore, the ICG-enema-based technique appears to also be effective in the identification of early, tiny proliferative lesions. Measurement of anti-cancer drug effect on the polyp growth in colon tumor models may be one of the potential applications of ICG fluorescence imaging. Furthermore, recently, TJ proteins also have been investigated as a target for cancer therapy [51]. We think that our technique may also be suitable to examine the cancer treatment efficacy of TJ targeting therapy.

In conclusion, our data demonstrate that fluorescence contrast-enhanced imaging following ICG administration by enema can be applied to colonoscopy for the detection of mucosal proliferative lesions. The tissue preference of ICG in the present model likely reflects the disruption of TJ structure, which in turn promotes the endocytic activity of proliferative cells and thus the cellular uptake of ICG.

## MATERIALS AND METHODS

### Fluorescent agents

Pharmaceutical grade ICG (Diagnogreen) was purchased from Daiichi-Sankyo (Tokyo, Japan). Blood vessels were visualized using *Lycopersicon esculentum* (tomato) lectin conjugated with DyLight 488 (TL488; Vector Laboratories, Burlingame, CA, USA).

### Fluorescence imaging instruments

Microscopic imaging was performed using a fluorescence reflectance imaging system (OV100; Olympus Corporation, Tokyo, Japan) and a multi-wavelength laser scanning microscope (IV100; Olympus) to acquire wide-field and high-power images, respectively [18]. Live cells and tissue sections were scanned using a fluorescence virtual microscopy system (VS120-FL; Olympus) [18]. Endoscopic imaging was performed using an in-house endoscopic system, as described previously [14]. ICG fluorescence was excited by a xenon light source through a 600 ± 200-nm band-pass filter and detected by a sensitive electron-multiplying charge-coupled device camera (MC285SPD-L0B0; Texas Instruments, Dallas, TX, USA) through a 842.5 ± 17.5-nm band-pass filter. As a flexible endoscope, a bronchoscope fiberscope (BF-XP60; Olympus Medical Corporation, Tokyo, Japan), 2.8 mm in diameter with a single biopsy channel, was used. Fluorescence images were shown in grayscale or pseudo-colors.

### Cell culture

The rat colon cancer cell line RCN-9 was obtained from the Japanese Collection of Research Bioresources (Osaka, Japan). RCN-9 was established from a rat colon adenocarcinoma induced in a F344 rat by the subcutaneous administration of the colon carcinogen DMH [41]. The cells were cultured in RPMI-1640 medium (Thermo Fisher Scientific, Carlsbad, CA, USA), supplemented with 10% fetal bovine serum, 100 U penicillin/ml, and 0.1 mg streptomycin/ml, at 37°C in a 5% CO_2_ humidified atmosphere.

### Live-cell imaging

Live-cell imaging was performed as described previously [18]. The experiments were performed using cells grown on non-coated chamber slides (81 mm^2^ per chamber) for 2 days after they were seeded at a concentration of 4 × 10^4^ cells/chamber. The cells were washed twice with Hank's buffered salt solution (HBSS), incubated with ICG (25 μM) in HBSS for 30 min at 37°C, washed twice in HBSS, incubated with Hoechst 33342 (Thermo Fisher Scientific) for 10 min at 37°C, and subjected to imaging analysis. To determine the intracellular localization of ICG, the cells were co-stained with the organelle markers listed in Supplementary Table 1. To investigate the contribution of endocytosis to ICG uptake, the cells were incubated with ICG at 4°C. The contribution of influx transporters to ICG uptake was evaluated in cells pre-incubated with 250 μM BSP (Sigma-Aldrich, St Louis, MO, USA), a competitive inhibitor of OATPs and NTCP, for 5 min, followed by incubation with ICG in the presence of the inhibitor. The mean fluorescence intensity (MFI) of ICG in whole-scan view (field-of-view ≈2–4 mm^2^) was measured using VS120-FL software. All cultured-cell experiments were performed in duplicate.

### Experimental tumor model

The 12 female F344 rats (4 weeks old) used in this study were purchased from Charles River Laboratories Japan (Kanagawa, Japan). They were fed CE-2 (CLEA Japan Inc, Tokyo, Japan) as a basal diet and provided with water ad libitum throughout the experimental period. Starting at 6 weeks of age, the rats were subcutaneously injected with AOM (Sigma-Aldrich; 15 mg/kg body weight) once a week for 3 weeks [52]. Animal experiments were performed 32–52 weeks after subcutaneous injection of the rats with AOM. Four animals were administered the ICG enema, four served as negative controls, and four were used in the immunofluorescence analysis of the colonic proliferative lesions. All animal experiments were performed in accordance with the guidelines for animal experimentation of the Facility of Agriculture, Tokyo University of Agriculture and Technology.

### *In vivo* and *ex vivo* fluorescence imaging

Prior to imaging, the rats were anesthetized through a nose cone with 2–3% isoflurane and placed in the supine position on a heated surgery pad. A flexible sonde and syringe were used to administer the ICG-containing enema. Four animals received phosphate-buffered saline (PBS) to clear the feces from the colon, followed by 10 mL (30 μg/mL) of ICG. Retention of the enema for 30 min was achieved by pinching the anus of the treated rats using a forceps. The concentration of ICG was determined based on the results of preliminary experiments using a dilution series of ICG and the cultured cells (Supplementary Figure 1). The volume of the enema was also based on the results of a previous study in which another fluorescence agent was administered to rats via enema [53]. TL488 was administered intravenously at 250 μg/250 μL per rat 20 min after ICG administration. After the 30-min ICG exposure, the rat intestinal tract was washed with PBS to remove the excess ICG. The rats were then euthanized by exsanguination under isoflurane anesthesia. The excised colon tissues were cut to reveal the intestinal mucosa, washed with PBS, and then subjected to *ex vivo* microscopic imaging. As a control, colon tumor tissues from four rats without ICG enema administration were used. For *in vivo* ICG fluorescence imaging, the endoscope was introduced intrarectally into anesthetized rats followed by the gentle insufflation of air. White-light and fluorescence images were acquired sequentially. To examine the effect of mucus on the ICG staining efficacy, WGA conjugated with Alexa Fluor 555 (Thermo Fisher Scientific) was used. Two mL of fluorescent WGA at 10 μg/mL was applied on the mucosal surface of the excised colon tissues and incubated for 15 min at room temperature, followed by PBS wash to remove the excess fluorescent WGA.

### Histology and immunofluorescence

In all animals, the excised colon tissues used in the histopathological examination were fixed overnight in phosphate-buffered 10% formalin (pH 7.4). Paraffin-embedded tissue sections stained with hematoxylin and eosin (HE) were used to diagnose the proliferative lesions in the rat colon. Tumors with tubular formation that invaded the submucosa were diagnosed as adenocarcinoma, and those with a tubular structure that did not invade the submucosa but compressed the surrounding crypts, as adenoma. Lesions that consisted of aggregations of dysplastic crypts were diagnosed as ACF. Small aggregations of tubular structures with no dysplasia were diagnosed as focal mucosal hyperplasia.

The tumor tissues from four rats were snap-frozen in liquid nitrogen for immunofluorescence analysis. Cryosections fixed with methanol were incubated first with the primary antibodies listed in Supplementary Table 2, then with species-appropriate Alexa Fluor-conjugated antibodies (Thermo Fisher Scientific), followed by counterstaining with 4′,6-diamidino-2-phenylindole, dilactate (Thermo Fisher Scientific).

## SUPPLEMENTARY MATERIALS FIGURES AND TABLES



## Abbreviations

ACF: aberrant crypt foci
AF: autofluorescence
AOM: azoxymethane
BSP: sulfobromophthalein
DMEM: Dulbecco's modified Eagle's medium
DMH: 1,2-dimethylhydrazine
HBSS: Hank's buffered salt solution
HE: hematoxylin and eosin
ICG: indocyanine green
MFI: mean fluorescence intensity
NTCP: sodium-taurocholate co-transporting polypeptide
OATP: organic anion transporting polypeptide
PBS: phosphate-buffered saline
TJ: tight junction
TL488: *Lycopersicon esculentum* (tomato) lectin conjugated with DyLight 488
WGA: wheat germ agglutinin
WL: white light
ZO-1: zona occludens-1
