# Supplementary material for: Fluorescence contrast-enhanced proliferative lesion imaging by enema administration of indocyanine green in a rat model of colon carcinogenesis
Source: Oncotarget. 2017 Oct 9;8(52):90278–90. doi: 10.18632/oncotarget.21744 (PMC5685748; doi:10.18632/oncotarget.21744)
Supplement: Supplementary file 1 [file oncotarget-08-90278-s001.pdf]

Fluorescence contrast-enhanced proliferative lesion imaging by enema administration of indocyanine green in a rat model of colon carcinogenesis

SUPPLEMENTARY MATERIALS

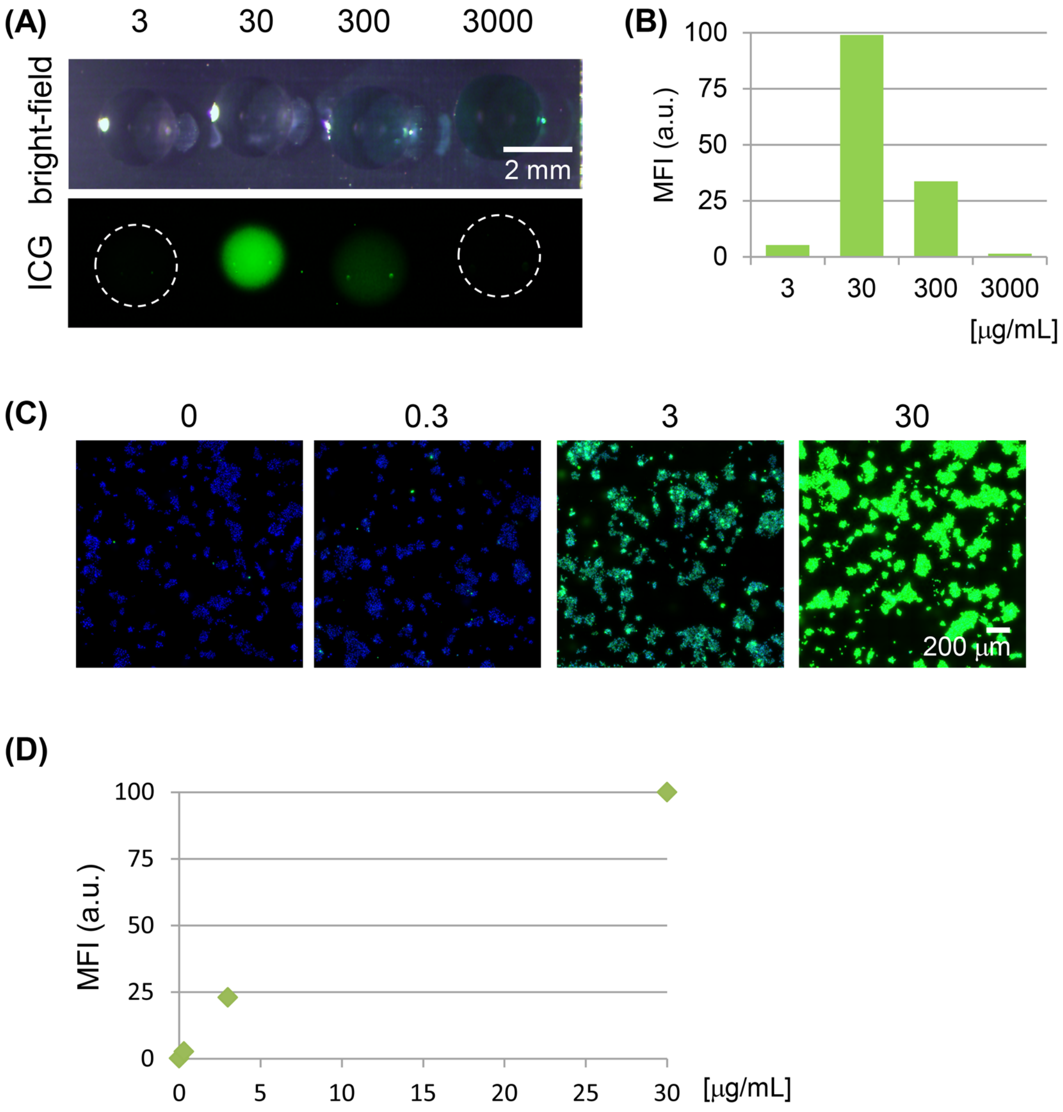

Supplementary Figure 1: Fluorescence intensity of an indocyanine green (ICG) dilution series. **(A)** Fluorescence imaging of ICG droplets at concentrations of 3, 30, 300, or 3000 μg/mL on a slide glass. ICG was dissolved in distilled water. Note that fluorescence quenching was observed at ICG concentrations of 300 and 3000 μg/mL. Scale bar, 2 mm. **(B)** Mean fluorescence intensity (MFI) of the ICG droplets. **(C)** Whole-scan view of live RCN-9 cells incubated with ICG (green) at concentrations of 0, 0.3, 3, or 30 μg/mL at 37°C for 30 min, followed by incubation with Hoechst 33342 (blue). Scale bar, 200 μm. **(D)** MFI of the ICG-labeled RCN-9 cells per whole-scan view.

Supplementary Table 1: List of fluorescent organelle markers used in this study

Organelle	Probes for organelle*	Manufacturer
Golgi complex	BODIPY FL C5-ceramide complexed to BSA	Thermo Fisher Scientific (Carlsbad, CA, USA)
Endoplasmic reticulum	ER-Tracker Green (BODIPY FL Glibenclamide)	Thermo Fisher Scientific
Mitochondria	MitoTracker Orange CMTMRos	Thermo Fisher Scientific
Lysosome	LysoTracker Red DND-99	Thermo Fisher Scientific
Nucleus	Hoechst 33342	Thermo Fisher Scientific

*Live-staining was carried out after ICG incubation and according to the manufacturer's instructions.

Supplementary Table 2: Antibodies used for immunofluorescence in this study

Antigen	Abbreviated name	Manufacturer	Product no.	Host species	Dilution
E-cadherin	—	BD Biosciences (San Jose, CA, USA)	610405	Mouse	1:10
Sodium-taurocholate co-transporting polypeptide	NTCP	Santa Cruz Biotechnology (Dallas, TX, USA)	sc-98484	Rabbit	1:50
Organic anion transporting polypeptide 1B2	OATP1B2 (OATP4)	Santa Cruz Biotechnology	sc-134461	Rabbit	1:50
Occludin	—	Thermo Fisher Scientific	40-4700	Rabbit	1:100
Zona occludens-1	ZO-1	Thermo Fisher Scientific	40-2200	Rabbit	1:100
